# Editorial: Robust, reliable, and continuous assessment in health: the challenge of wearable and remote technologies

**DOI:** 10.3389/fphys.2023.1281426

**Published:** 2023-09-12

**Authors:** Jordi Aguiló, Driss Moussaoui, Ki Chon, Raquel Bailón

**Affiliations:** ^1^ Centro de Investigación Biomédica en Red, Madrid, Spain; ^2^ Universitat Autònoma de Barcelona, Barcelona, Spain; ^3^ Ibn Rushd University Psychiatric Centre, Casablanca, Morocco; ^4^ University of Connecticut, Storrs, CT, United States; ^5^ Universidad de Zaragoza, Zaragoza, Spain

**Keywords:** medical diagnosis, continuous monitoring, physiological data, emotional and cognitive assessment, healthcare innovation, wearable technology, personalized medicine

Medical diagnosis has always relied on knowledge, technology, and creativity, as well as the ability to interpret vast amounts of diverse data to facilitate disease detection and improve patient outcomes. Medical diagnosis involves drawing quantitative conclusions from various physiological data, including skin temperature (ST), heart rate (HR), respiratory rates (RR), blood pressure (BP), and many more. Other physiological parameters such as skin color, facial expression, voice or gait characteristics can also provide some additional information. Furthermore, information about the individual’s mental state, such as stress levels, emotional states, or symptoms of mental disorders, could also add a holistic perspective to the diagnostic process.

The challenge of an accurate medical diagnosis lies in dealing with heterogeneous data with different granularity and reliability degrees, missing data, and subjective or unquantifiable data. Healthcare researchers should analyze unstructured information from patients with similar symptoms to draw general principles that, when validated, could contribute to create new knowledge. Wearable technologies, based on ECG and PPG acquisition, allow the quantification of HR changes throughout the entire day as the subject moves freely. As such, this information can be used for screening and monitoring arrythmias and other cardiovascular diseases, but also metabolic, respiratory and even mental disorders.

While more substantial evidence is needed, it is clear that emotional state has an influence on physical state and *vice versa*. It is essential to incorporate the emotional state into the diagnostic process and the prescription of treatments. However, assessing emotional information is challenging, as it relies heavily on the observer, and quantifying emotion accurately is complex. Recent advances using machine and deep learning on electrodermal activity combined with HRV data have shown promising potential for quantitative assessment of emotional information. Anticipating the rise of personalized medical diagnosis, technology is adapting to the new possibilities and wearable devices are evolving in this direction.

Today, almost all wearables offer HR features extracted from photoplethysmographic signals (PPG), that can provide information about stress and mood. PPG combined with ECG can also provide estimates of arterial pressure although they have not been proven to be accurate enough to meet medical quality standards. These physiological and mental parameters might still offer the potential for personalized medical diagnosis or, at least, triggering alerts and professional reviews.

In the near future, the technology will incorporate new sensors, better algorithms Aided by advances in artificial intelligence having easy access to vast databases.

All these novelties will undoubtedly improve the reliability of most physiological and emotional variables in use what, in turn, could significantly enhance diagnostic accuracy and provide valuable support to healthcare professionals. Moreover, wearable devices and smartphones are likely to converge in a digital environment, allowing continuous monitoring of various aspects of an individual’s health in a transparent manner.

On this specific Research Topic, several papers have been published addressing the various medical applications mentioned above and focusing on the intersection of technology and healthcare. Ryu et al., explored the use of telemedicine in combination with wearable devices to facilitate remote medical diagnosis in space or rural areas by introducing remote auscultation, percussion, and palpation.

Another study from Väliaho et al., demonstrated that precise analysis of a good quality PPG signal registered during 24 h enables the detection of asymptomatic atrial fibrillation, a significant risk factor for stroke in the elderly population.

Additionally, in Psychosomatic response to acute emotional stress in healthy students, García et al. Have quantitatively assessed induced acute stress in a healthy students population by using electrophysiological variables and developed mathematical models to enhance stress evaluation. Models are then validated against gold standard test.

Moreover, wearable sensors have proven reliable in quantifying fatigue and sleep, providing objective data for disease surveillance and evaluating treatment outcomes as it is published by Antikainen et al. Assessing fatigue and sleep in chronic diseases using wearables. Further, wearables have enabled the assessment of physical rehabilitation outcomes through unobtrusive monitoring such as an unintentional walk test in free-living activities as it is shown by Sokas et al.

Kontaxis et al. Demonstrates that autonomic function, as measured through HRV by using a wearable device, is a useful way to assess clinical outcomes in multiple sclerosis patients, and also to monitor the severity of the disorder.

Furthermore, Molinaro et al. have investigated contactless estimation of physiological variables through recorded videos obtained through smartphones or webcam cameras, extending data capture to respiratory, cardiac parameters, SpO2, and blood pressure.

Regarding therapies to assess and to mitigate mental health symptoms, Ribeiro et al., are proposing a multiparameter model based on electrophysiological signals to evaluate the effectiveness of interventions such as biofeedback and mindfulness. Proposed models are providing relevant information about current mental health of the individual which is useful to objectively assess the effectiveness of stress reduction interventions.

In conclusion, the combination of knowledge, technology, and creativity promises to significantly enhance medical diagnosis and therapies. The increasing popularity of wearable technology such as connected watches and rings, with over 100 million units sold worldwide each year, equipped with advanced sensors that generate vast amounts of data, coupled with advancements in artificial intelligence, has the potential to change the healthcare paradigm.

These devices will offer continuous monitoring and objective assessment of numerous physiological and emotional variables that will process to promote physical exercise and sports as preventive and therapeutic measures against various health conditions such as obesity, diabetes, high blood pressure, cardiovascular diseases, and cancer. Additionally, they can play a preventive and curative role in addressing stress, anxiety disorders, depressive disorders, and Alzheimer’s disease.

The implication of this technological advancement is huge from a public health perspective. The increased accuracy in diagnosis and improved therapies will benefit both healthcare professionals and patients alike. It represents a promising step forward in the field of healthcare, empowering individuals to take a proactive approach to their wellbeing and potentially leading to better overall health outcomes.

